# Genome Sequence of *Sphingomonas xenophaga* QYY, an Anthraquinone-Degrading Strain

**DOI:** 10.1128/genomeA.00031-12

**Published:** 2013-01-15

**Authors:** Yuanyuan Qu, Xuwang Zhang, Hao Yu, Hongzhi Tang, E Shen, Hao Zhou, Qiao Ma, Xiangyu Cao, Jiti Zhou, Ping Xu

**Affiliations:** a State Key Laboratory of Fine Chemicals and Industrial Ecology and Environmental Engineering, School of Environmental Science and Technology, Dalian University of Technology, Dalian, China; b State Key Laboratory of Microbial Metabolism and School of Life Sciences and Biotechnology, Shanghai Jiao Tong University, Shanghai, China

## Abstract

*Sphingomonas xenophaga* QYY is an efficient anthraquinone-degrading strain. Here, we present a 4.2-Mb assembly of the first genome sequence of *S. xenophaga*. We have annotated 36 coding sequences (CDSs) encoding aromatic catabolism and 216 CDSs responsible for toxic resistance and stress response, which may provide insights into the degradation of complex aromatics.

## GENOME ANNOUNCEMENT

The strains of the genus *Sphingomonas* have been paid much attention due to their versatile capabilities to degrade various pollutants, including polycyclic aromatic hydrocarbons and other aromatic contaminants. They play important roles in biotechnological and industrial applications (1, 2). Compared with other species, *Sphingomonas xenophaga* has the potential to detoxify anthraquinones (2). We have previously reported that the *S. xenophaga* strain QYY was proven to efficiently degrade an anthraquinone compound, bromoamine acid (BAA), in BAA wastewater treatment systems (3–5). This is the first report of a draft genome sequence of an *S. xenophaga* strain associated with anthraquinone degradation. The *S. xenophaga* genome sequence may provide novel molecular information to reveal the degradation mechanisms of anthraquinones or other complex aromatics by *Sphingomonas xenophaga* strain QYY.

The draft genome sequence of *Sphingomonas xenophaga* strain QYY was obtained using Solexa paired-end sequencing with a HiSeq 2000 system (100 bp for each read). The reads were assembled to 129 large contigs using Velvet software (6). The largest contig assembled was approximately 289 kb (the *N*_50_ was approximately 89 kb). Gene prediction and genome annotation were carried out using the RAST autoannotation server and the NCBI PAPPC pipeline (7, 8). The tRNA was predicted using tRNAscan software (9). The gene function and classification were determined using the KEGG and Clusters of Orthologous Groups (COG) databases (10).

The genome sequence of strain QYY is 4,221,110 bp in length, with a G+C content of 63.1%. The genome encodes 3,988 putative coding sequences (CDSs) (927-bp average length, 87.6% coding density), of which only 1,547 CDSs (38.7%) have functional predictions. The genome of strain QYY contains 3 ribosomal operons and 49 tRNA loci. There are 394 subsystems represented in the genome sequence (1,837 CDSs in total), and the metabolic network of strain QYY (determined by the RAST server) was reconstructed (7). We have predicted a rich set of genes (36 CDSs) responsible for the degradation of aromatic compounds, which should be related to the metabolism of BAA. Meanwhile, there are 75 CDSs that were annotated as the genes for resistance to antibiotics and toxic compounds, 299 CDSs for carbohydrate metabolism, and 141 CDSs for the stress response. All of these genes may contribute to bioaugmentation in polluted water. However, for 1,450 CDSs the functions were not predicted, and 389 genes had no homologous gene found in any of the released genome sequences of other *Sphingomonas* strains (using tBLASTn, e < 10^–5^). All of these results reveal that the strain QYY has a diverse catabolic ability and may contain many new gene resources for metabolism and bioaugmentation (11, 12). Only 12 genome sequences of the *Sphingomonas* strains were released, and no genome sequences of *Sphingomonas xenophaga* species have been previously published. The genomic information about this genus with the genome sequence of strain QYY provides new insights into the genetic versatility of *Sphingomonas* strains and demonstrates the metabolism of complex aromatics.

### Nucleotide sequence accession numbers. 

This whole-genome shotgun project has been deposited at DDBJ/EMBL/GenBank under the accession number AKIB00000000. The version described in this paper is the first version, AKIB01000000.
